# Temperature Stability of a Hybrid Polarization-Maintaining Photonic Crystal Fiber Resonator and Its Application in a Resonant Fiber Optic Gyro

**DOI:** 10.3390/s18082506

**Published:** 2018-08-01

**Authors:** Nie He, Zhuoyan Li, Gongshen Zhang, An’an Liu, Ding Zhou, Peng Chen, Chengxiang Liu, Xu Wu

**Affiliations:** 1 Guangdong Provincial Key Laboratory of Micro/Nano Optomechatronics Engineering, College of Mechatronics and Control Engineering, Shenzhen University, Shenzhen 518060, China; 2160190401@email.szu.edu.cn (N.H.); 2160190403@email.szu.edu.cn (Z.L.); 2172291807@email.szu.edu.cn (A.L.); 2172291838@email.szu.edu.cn (P.C.); 2 Guangdong Provincial Key Laboratory of Micro/Nano Optomechatronics Engineering, College of Optoelectronic Engineering, Shenzhen University, Shenzhen 518060, China; 2161190230@email.szu.edu.cn (G.Z.); 2170285224@email.szu.edu.cn (D.Z.); 3 Sino-German College for Intelligent Manufacturing, Shenzhen Technology University, Shenzhen 518060, China

**Keywords:** resonant fiber optic gyro, polarization fluctuation, polarization-maintaining photonic crystal fiber

## Abstract

A fiber ring resonator (FRR) constructed using a Panda polarization-maintaining fiber does not effectively solve the problem of temperature-related polarization fluctuation, which considerably limits the detection accuracy of the resonant fiber optic gyro. The polarization-maintaining photonic crystal fiber (PM-PCF) can improve the thermal stability of the FRR. In this study, a structure that can simultaneously detect the polarization fluctuation of two FRRs is designed. We analyzed and verified the polarization phase shift errors of these two types of fibers, which are caused by the thermally induced birefringence changes. Theoretical simulation and experimental results confirm that a PM-PCF can be used to optimize the FRR, which can effectively suppress the polarization fluctuation.

## 1. Introduction

A gyro is an inertial sensor to measure the rotation rate [1]. A resonant fiber optic gyro (RFOG) based on the Sagnac effect exhibits the advantages of high sensitivity and large dynamic range [2]. When compared with the traditional interferometric fiber optic gyro (IFOG), an RFOG is observed to achieve a similar detection precision but with a much shorter fiber length, providing superiority with respect to its further integration and augmenting its application value in the fields of navigation, aerospace, defense, and guidance systems [1,2,3,4,5,6].

A fiber ring resonator (FRR) is used as the sensitive ring in an RFOG [7]. Multibeam interference in the FRR is used to enhance the optical Sagnac effect that is caused by rotation; thus, the performance of the FRR directly affects the detection accuracy of an RFOG. There are several factors that degrade the RFOG performance such as polarization fluctuation, backscatter noise, and the optical Kerr effect. Among the various factors, temperature-related polarization fluctuation is considered to be the main reason for the long-term instability of an RFOG [8,9,10,11,12,13,14,15]. Generally, two eigenstates of polarization (ESOPs), which are the states of polarization (SOPs) that are capable of returning to their original states after one-turn propagation through the FRR, are simultaneously excited in the resonator [8]. The desired ESOP, ESOP1, is used to detect the rotation of the system. The undesired ESOP, ESOP2, drifts through the desired ESOP as the temperature of the FRR varies, thus degrading the gyro performance [13,16]. Various research groups have proposed different resonator schemes to overcome polarization-fluctuation, such as a single 90° polarization-axis rotated splice, twin 90° polarization-axis rotated splices, construction of a hybrid single-polarization FRR, and the use of a polarization controller [9,10,11,12,16,17,18,19,20,21,22,23,24,25,26,27,28,29,30].

A conventional polarization maintaining fiber (PMF), which typically exhibits high birefringence [31], can be used to construct an FRR in a RFOG. Common PMFs, such as the Panda-PMF, exhibit two circular stress applying parts (SAPs) on both sides of their core, thereby increasing the birefringence of the fiber. This makes the propagation constants of the two ESOPs markedly different, and coupling does not readily occur, thus maintaining the polarization. However, there are several kinds of media in the Panda-PMF. Certain physical properties of these media, such as the refractive index and heat transfer rate, are considerably affected by the temperature, which exacerbates the crosstalk between the two ESOPs of an FRR. An FRR constructed using a Panda-PMF does not effectively solve the problem of temperature instability, which introduces severe polarization crosstalk and considerably limits the detection accuracy of an RFOG [32].

In contrast, a photonic crystal fiber (PCF) exhibits much better temperature stability [33,34,35,36], which can effectively solve the problem of thermal instability [31]. This is because the cladding in PCF contains multiple air holes that confine light within the core and because the thermal constant of air is much lower than that of silica. Recently, PCFs with an extensive range of designs, including a polarization-maintaining photonic crystal fiber (PM-PCF) have become available. Generally, a PM-PCF is based on the original PCF but contains air holes of increased sizes on both sides of the core, thereby increasing the birefringence. Further, a PM-PCF can maintain polarization and improve the thermal stability. The undesirable thermal effects of conventional PMF can be considerably reduced by replacing the conventional PMF in an RFOG system with a PM-PCF [33,37,38,39,40,41,42].

In this study, for Panda-PMF and PM-PCF, we theoretically analyzed and experimentally verified the polarization phase shift errors caused by the thermally induced birefringence changes. Two couplers with Panda-PMF pigtails were fused to a section of Panda-PMF and a section of PM-PCF to construct two FRRs, i.e., FRR1 and FRR2. FRR2 was a hybrid FRR comprised of two types of optical fibers. The Jones matrix method was used to theoretically analyze the thermal stability of the polarization of the two FRRs. The birefringent temperature coefficients of Panda-PMF and PM-PCF were calculated using the finite element numerical calculation method. Experimentally, we designed a structure that can simultaneously detect the temperature-related polarization fluctuation of two FRRs. We measured the polarization crosstalk periods of the FRRs. Further, the birefringent temperature coefficients of the two types of fibers were calculated and compared with the experimental results. Both the theoretical simulation and the experimental results confirmed that a PM-PCF can be used to optimize an FRR, which can effectively suppress the polarization fluctuation.

## 2. System Structure of the RFOG

**Figure 1:**
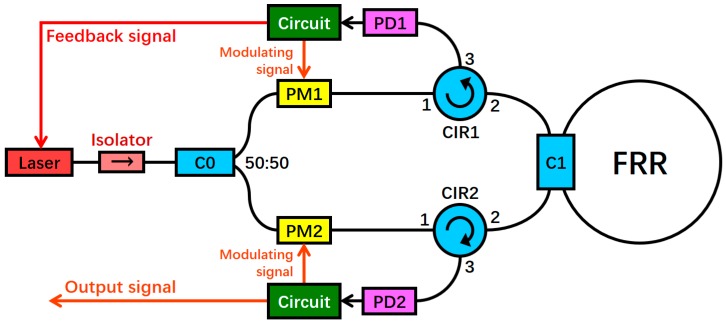
System structure of the resonant fiber optic gyro (RFOG) (PD: photoelectric detector, PM: phase modulator, C: coupler, CIR: circulator, and FRR: fiber ring resonator).

The RFOG system structure is depicted in Figure 1. The laser light emitted by a narrow-line-width semiconductor laser is divided into two equal power beams by a 50:50 coupler C0 after passing through an isolator. The two light beams pass through the LiNbO_3_ phase modulators (PMs), PM1 and PM2, which are modulated by signals with different frequencies. Subsequently, the two beams are incident to the FRR through the circulators (CIRs), i.e., CIR1 and CIR2, in the counterclockwise (CCW) and clockwise (CW) directions, respectively. C1 is the coupler that is connected to the FRR. Finally, the two light beams are provided as output to the photoelectric detectors (PDs), PD2 and PD1, via CIR2 and CIR1, respectively. Among the light waves, the CW light wave is processed by a digital detection control circuit based on a field-programmable gate array (FPGA). Subsequently, the circuit outputs feedback signals to control the center frequency of the laser; thus, the laser is locked into the resonant frequency of the CW light wave. At this moment, the output optical signal of the CCW light wave can reflect the resonant frequency difference of the CW and CCW light waves. Furthermore, the rotational angular speed can be obtained after being processed by the circuit [3].

## 3. Theoretical Analysis and Simulation Results

### 3.1. Theoretical Analysis of Polarization Fluctuation in FRR

An FRR is used as the sensitive ring in an RFOG [7]. Multibeam interference in an FRR is used to enhance the optical Sagnac effect caused by rotation; thus, the performance of an FRR directly affects the detection accuracy of an RFOG. Conventional PMF, such as Panda-PMF, which typically has high birefringence, can be used to construct an FRR in an RFOG [31]. However, there are several kinds of media in a Panda-PMF, including an acrylate coating, cladding (SiO_2_ material), core (SiO_2_ + GeO_2_), and two SAPs (SiO_2_ + B_2_O_3_). Some of the physical properties of these media, such as the refractive index and heat transfer coefficient, are markedly affected by temperature. The mismatch in the thermal expansivity coefficient among the different media results in thermal stress among the structures, and the stress alters the effective mode refractive index through the elasto-optic effect. Furthermore, the material refractive indices change due to temperature changes (thermo-optical effects) of different media [43]. The above factors exacerbate the crosstalk between the two ESOPs of an FRR. To overcome this shortcoming, a PM-PCF can suppress the polarization fluctuation well. Because the PM-PCF contains only one kind of material (SiO_2_) inside the coating, the thermal stress and elasto-optic effects are small. Additionally, there are multiple air holes in a PM-PCF, and the physical properties of air, such as the refractive index, are less affected by temperature. Therefore, splicing a section of a PM-PCF into an FRR can reduce the influence of polarization crosstalk. It is expected to improve the precision of the angular velocity measurement.

**Figure 2:**
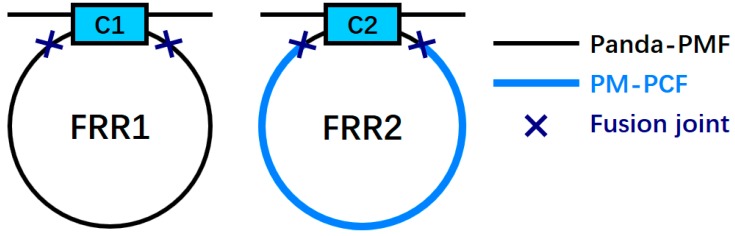
Structures of two FRRs (FRR: fiber ring resonator, Panda-PMF: Panda polarization maintaining fiber, PM-PCF: polarization-maintaining photonic crystal fiber, C: coupler).

As depicted in Figure 2, we constructed two FRRs in which FRR1 was formed by fusing a section of Panda-PMF and coupler C1, and FRR2 was formed by fusing a section of a PM-PCF and coupler C2. The pigtails of the two couplers were both constructed from a Panda-PMF. The remaining basic parameters of both couplers in the test sheets were similar. *β_x_* and *β_y_* are the propagation constants of the slow axis and fast axis polarization modes of a Panda-PMF, respectively; *β_x_*′ and *β_y_*′ denote the same, respectively, for a PM-PCF. The propagation constant differences caused by birefringence can be defined as Δ*β* = *β_x_* − *β_y_* and Δ*β*′ = *β_x_*′ − *β_y_*′. Further, the birefringences of a Panda-PMF and a PM-PCF can be respectively defined as Δ*n*_eff_ = *n*_eff,*x*_ − *n*_eff,*y*_ and Δ*n*′_eff_ = *n*′_eff,*x*_ − *n*′_eff,*y*_, where *n*_eff,*x*_ and *n*_eff,*y*_ are the effective mode indices of the slow and fast axis polarization modes of a Panda-PMF, whereas *n*′_eff,*x*_ and *n*′_eff,*y*_ denote the same in case of a PM-PCF, respectively. According to the relation between the propagation constant and the effective mode refractive index, we can obtain

(1) $$\Delta \beta =k_{0}\Delta n_{\mathrm{eff}},$$

(2) $$\Delta \beta^{\prime }=k_{0}\Delta n_{\mathrm{eff}}^{\prime },$$

where *k*_0_ is the wave number of light in vacuum.

Subsequently, we theoretically analyze the influence of temperature on the two FRRs. The total lengths of the two FRRs remain equal and are both *L*. The optical power coupling ratio between the coupler direct port and cross port is *k*:(1 − *k*). The remaining percentage of optical power that is caused by insertion loss of the coupler is *ρ*_c_ [3].

#### 3.1.1. Phase Variation Difference of the Two ESOPS of FRR1

For FRR1, the total loss coefficient of all the fusion joints is α*_R_*. The average transmission loss coefficient of the fiber is *α_L_*. Further, the Jones matrix of the entire fiber in FRR1 is

(3) $$F_{1}=e^{-\alpha_{R}}\left(\begin{array}{cc} e^{-\left(\alpha_{L}-j\beta_{x}\right)L} & 0\\ 0 & e^{-\left(\alpha_{L}-j\beta_{y}\right)L} \end{array}\right).$$

Therefore, the matrix in FRR1 after one-turn propagation is

(4) $$R_{1}=\sqrt{k\rho_{c}}F_{1}.$$

The two eigenvalues of the aforementioned matrix are

(5) $$\zeta_{1x}=\sqrt{k\rho_{c}}e^{-\alpha_{R}-\alpha_{L}L+j\beta_{x}L},$$

(6) $$\zeta_{1y}=\sqrt{k\rho_{c}}e^{-\alpha_{R}-\alpha_{L}L+j\beta_{y}L}.$$

These correspond to the two ESOPs of FRR1. Therefore, after one-turn propagation, the phase variations of the two ESOPs are

(7) $$\Phi_{1x}=\arg\left(\zeta_{1x}\right)=\beta_{x}L,$$

(8) $$\Phi_{1y}=\arg\left(\zeta_{1y}\right)=\beta_{y}L.$$

The phase variation difference between the two ESOPs is

(9) $$\Delta \Phi_{1}=\Phi_{1x}-\Phi_{1y}=\Delta \beta L.$$

From Equations (1) and (9), it can be observed that Δ*Φ*_1_ is related to the birefringence, Δ*n*_eff_, of a Panda-PMF.

#### 3.1.2. Phase Variation Difference of the Two ESOPS of FRR2

For FRR2, a section of PM-PCF is fused to the ring. The length of a PM-PCF is *L*′; the total length of hybrid FRR2 remains as *L*. The total loss coefficient of all the fusion joints is *α_R_*′. The average transmission loss coefficient of a PM-PCF is *α_L_*′. Further, the Jones matrix of the entire fiber in FRR2 is

(10) $$F_{2}=e^{-\alpha_{R}}\left(\begin{array}{cc} e^{-\left(\alpha_{L}-j\beta_{x}\right)\left(L-L^{\prime }\right)-\left(\alpha_{L}^{\prime }-j\beta_{x}^{\prime }\right)L^{\prime }} & 0\\ 0 & e^{-\left(\alpha_{L}-j\beta_{y}\right)\left(L-L^{\prime }\right)-\left(\alpha_{L}^{\prime }-j\beta_{y}^{\prime }\right)L^{\prime }} \end{array}\right).$$

Therefore, the matrix in FRR2 after one-turn propagation is

(11) $$R_{2}=\sqrt{k\rho_{c}}F_{2}.$$

The two eigenvalues of the aforementioned matrix are

(12) $$\zeta_{2x}=\sqrt{k\rho_{c}}e^{-\alpha_{R}-\left(\alpha_{L}-j\beta_{x}\right)\left(L-L^{\prime }\right)-\left(\alpha_{L}^{\prime }-j\beta_{x}^{\prime }\right)L^{\prime }},$$

(13) $$\zeta_{2y}=\sqrt{k\rho_{c}}e^{-\alpha_{R}-\left(\alpha_{L}-j\beta_{y}\right)\left(L-L^{\prime }\right)-\left(\alpha_{L}^{\prime }-j\beta_{y}^{\prime }\right)L^{\prime }}.$$

These correspond to the two ESOPs of FRR2. Therefore, after one-turn propagation of the two ESOPs in FRR2, the phase variations of the two ESOPs are

(14) $$\Phi_{2x}=\arg\left(\zeta_{x}\right)=\beta_{x}\left(L-L^{\prime }\right)+\beta_{x}^{\prime }L^{\prime },$$

(15) $$\Phi_{2y}=\arg\left(\zeta_{y}\right)=\beta_{y}\left(L-L^{\prime }\right)+\beta_{y}^{\prime }L^{\prime }.$$

The phase variation difference between the two ESOPs is

(16) $$\Delta \Phi_{2}=\Phi_{2x}-\Phi_{2y}=\Delta \beta \left(L-L^{\prime }\right)+\Delta \beta^{\prime }L^{\prime }.$$

From Equations (1), (2) and (16), it can be observed that Δ*Φ*_2_ is related to the birefringences, Δ*n*_eff_ and Δ*n*′_eff,_ of the two kinds of fibers.

#### 3.1.3. Comparison of Polarization Crosstalk Periods of the Two FRRs

From Equations (9) and (16), the phase variation differences per unit temperature change between the two ESOPs of FRR1 and FRR2 are observed to change by

(17) $$\frac{d\Delta \Phi_{1}}{dT}=\frac{d\Delta \beta }{dT}L,$$

(18) $$\frac{d\Delta \Phi_{2}}{dT}=\frac{d\Delta \beta }{dT}\left(L-L^{\prime }\right)+\frac{d\Delta \beta^{\prime }}{dT}L^{\prime },$$

where *T* is the temperature. Combining Equations (1), (2), (17) and (18), the polarization crosstalk periods of the two FRRs are

(19) $$\frac{2\pi dT}{d\Delta \Phi_{1}}=\frac{\lambda_{0}}{\frac{d\Delta n_{\mathrm{eff}}}{dT}L},$$

(20) $$\frac{2\pi dT}{d\Delta \Phi_{2}}=\frac{\lambda_{0}}{\frac{d\Delta n_{\mathrm{eff}}}{dT}\left(L-L^{\prime }\right)+\frac{d\Delta {n^{\prime }}_{\mathrm{eff}}}{dT}L^{\prime }},$$

where *λ*_0_ = 2π/*k*_0_.

To compare the temperature sensitivity of the two FRRs, the ratio of the polarization crosstalk periods of the two FRRs can be calculated as follows:

(21) $$\frac{\frac{d\Delta \Phi_{2}}{dT}}{\frac{d\Delta \Phi_{1}}{dT}}=1-\left(1-\frac{\frac{d\Delta {n^{\prime }}_{\mathrm{eff}}}{dT}}{\frac{d\Delta n_{\mathrm{eff}}}{dT}}\right)\frac{L^{\prime }}{L}.$$

It can be observed that the ratio of the polarization crosstalk periods is related to not only the ratio of the birefringence temperature coefficients of the two fibers but also to the ratio of the lengths of the two kinds of fibers.

### 3.2. Multiphysics Finite Element Modeling

**Figure 3:**
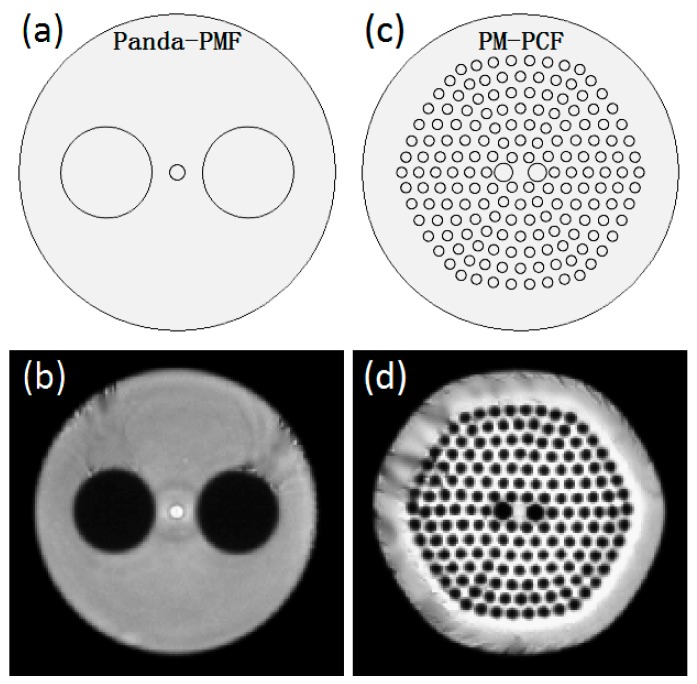
(**a**) Simulation geometry and (**b**) actual structure of the Panda-PMF; (**c**) simulation geometry and (**d**) actual structure of PM-PCF (Panda-PMF: Panda polarization maintaining fiber and PM-PCF: polarization-maintaining photonic crystal fiber).

To compare the birefringent temperature coefficients of the two fibers, the birefringent temperature characteristics of the two fibers can be characterized using a multiphysics finite element modeling method [31,37,43]. According to the actual structure of the cross-section end-faces of the two types of optical fibers (Figure 3b,d), the geometrical parameters were measured. These parameters are presented in Table 1. According to these parameters, the geometric structures were drawn, as depicted in Figure 3a,c. In these models, all the material parameters of the two types of optical fibers were set according to Table 2.

At different temperatures, the various materials in the fibers exhibited varying degrees of thermal expansion, and the stress that was generated among them eventually caused the slight changes in shape. Therefore, the refractive indices of various materials have changed, and the calculated effective mode refractive indices of the two ESOPs have also changed.

**Figure 4:**
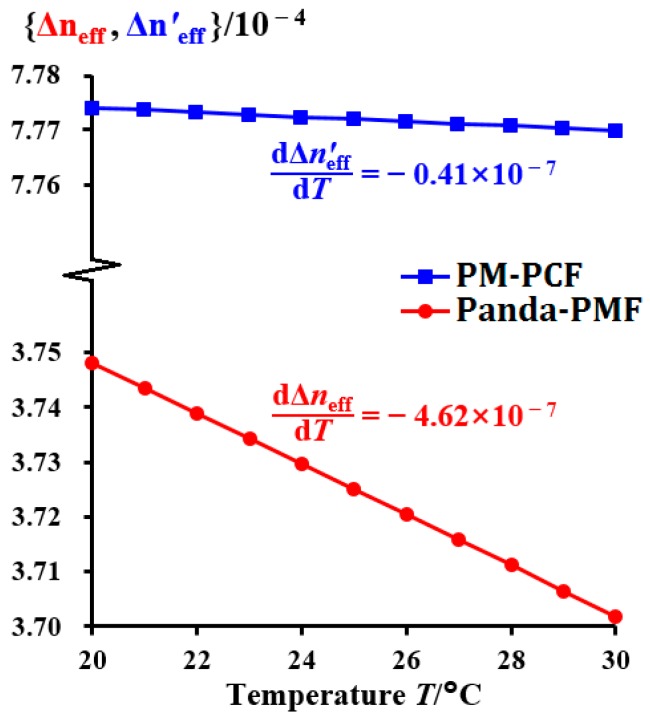
Birefringence simulation results of a Panda-PMF and a PM-PCF in the range of 20 °C to 30 °C (Panda-PMF: Panda polarization maintaining fiber and PM-PCF: polarization-maintaining photonic crystal fiber).

The birefringent temperature coefficients can be denoted as dΔ*n*_eff_/d*T* and dΔ*n*′_eff_/d*T*. As depicted in Figure 4, after multiphysics finite element calculations, dΔ*n*_eff_/d*T* = −4.62 × 10^−7^ and dΔ*n*′_eff_/d*T* = −0.41 × 10^−7^. Therefore, the simulation results exhibit that the temperature stability of a PM-PCF is 11.268 times that of a Panda-PMF. The birefringent temperature coefficients of the two fibers satisfy |dΔ*n*′_eff_/d*T*| < |dΔ*n*_eff_/d*T*|. Therefore, from Equation (21), it can be observed that |dΔ*Φ*_2_/d*T*| < |dΔ*Φ*_1_/d*T*| always holds; thus, the phase variation difference, Δ*Φ*_2,_ of the two ESOPs in FRR2 is less affected by temperature. Furthermore, the larger the value of *L*′/*L*, the smaller will be the value of |dΔ*Φ*_2_/d*T*|/|dΔ*Φ*_1_/d*T*|, which indicates that the greater the proportion of the length, *L*′, of a PM-PCF in the total ring length, *L*, of the entire FRR, the smaller will be the influence of temperature on the polarization fluctuation. Therefore, fusing a section of a PM-PCF in an FRR can theoretically reduce the effect of polarization crosstalk.

## 4. Experiment

### 4.1. Experiment System

**Figure 5:**
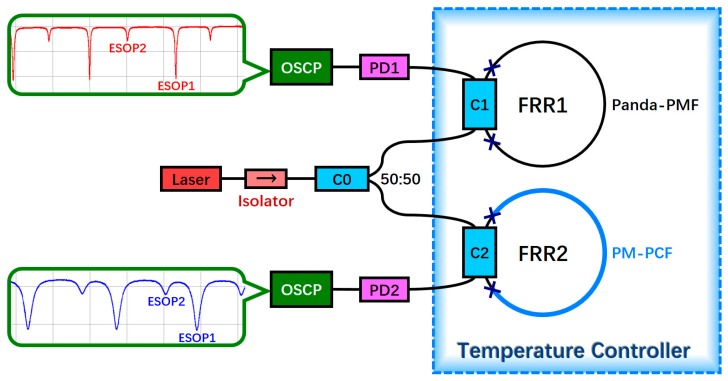
Structure that can simultaneously detect the temperature-related polarization fluctuation of two FRRs (ESOP: eigenstates of polarization, FRR: fiber ring resonator, OSCP: oscilloscope, Panda-PMF: Panda polarization maintaining fiber, PM-PCF: polarization-maintaining photonic crystal fiber, PD: photoelectric detector, and C: coupler).

As depicted in Figure 5, we designed a structure that can simultaneously detect the temperature-related polarization fluctuation of the two FRRs. The laser light emitted by a narrow-line-width semiconductor laser is divided into two equal power beams by a 50:50 coupler C0 after passing through an isolator. Subsequently, the two beams are incident to two FRRs. According to Equations (9) and (16), the lengths of the resonators should not be too large to study the temperature characteristics of the two types of fibers. The polarization characteristics of a Panda-PMF are considerably affected by the temperature; therefore, if the resonator length is very long, dΔ*Φ*_1_/d*T* is observed to be considerably large. When the temperature was slightly altered, the polarization crosstalk became a serious issue, which made the crosstalk period of FRR1 difficult to observe. FRR1 was formed by fusing a section of a Panda-PMF and coupler C1, whereas FRR2 was formed by fusing a section of a PM-PCF and coupler C2. The pigtails of the two couplers were constructed from a Panda-PMF. The total length of the resonator of each FRR was 4 m. The length of a PM-PCF in FRR2 was 3 m; thus, FRR2 was a hybrid FRR with a 3:1 ratio of the length of a PM-PCF to that of a Panda-PMF. To detect the effect of temperature on two FRRs, only two FRRs were placed inside the temperature controller, with the other optical devices placed outside. The output ports of the two FRRs were connected to PD1 and PD2. The two PDs were connected to the circuit. Their signals were monitored in real time using an oscilloscope (OSCP). By changing the frequency of the laser, the resonant curves of the two FRRs could be observed. Comparing these two resonant curves, it can be observed that the fineness of the curve of FRR2 is smaller than that of FRR1. This is mainly due to the fact that FRR2 is a hybrid resonator and the light field distributions of a PM-PCF and Panda-PMF are inconsistent, resulting in greater loss at the fusion joints of FRR2 than that observed at the fusion joints of FRR1. However, the above theoretical analysis and simulation results show that with the improvement of fiber fusion technology, a PM-PCF exhibits considerable potential in relation to FRR construction [44].

### 4.2. Experimental Results and Discussion

**Figure 6:**
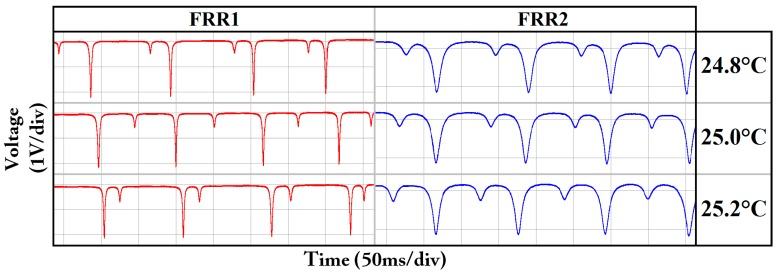
Resonant curves of the two FRRs at 24.8 °C, 25.0 °C, and 25.2 °C (FRR: fiber ring resonator).

The two FRRs were placed in a temperature controller. The objective of the temperature controller was to increase the temperature by 0.1 °C every 1.8 minutes, from 20 °C to 30 °C. The polarization crosstalk periods of the two FRRs were observed based on the resonant curves. Figure 6 depicts the resonant curves of two FRRs at approximately 25 °C. It can be observed that the crosstalk period of the two ESOPs in FRR2 is significantly longer than those of FRR1.

**Figure 7:**
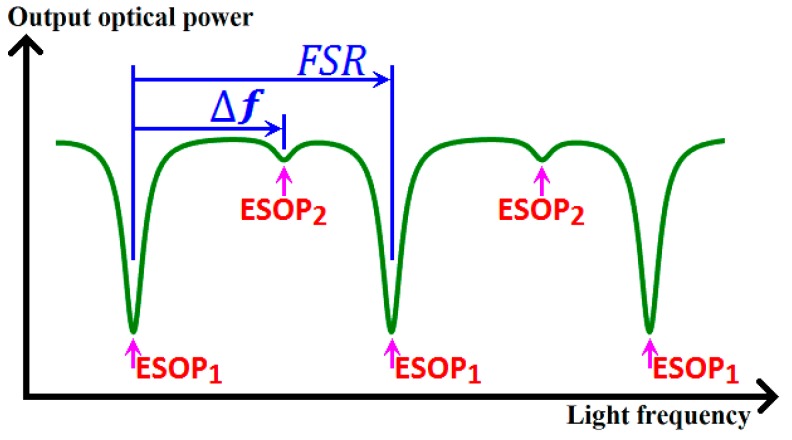
Resonant frequency difference Δ*f* between the two ESOPs and free spectral range (ESOP: eigenstates of polarization, and FSR: free spectral range).

As depicted in Figure 7, the phase variation difference Δ*Φ*, the resonant frequency difference Δ*f* between the two ESOPs, and the free spectral range (FSR) satisfy the following relation:

(22) $$\Delta \Phi =2\pi \cdot \left(\frac{\Delta f}{FSR}+m\right),$$

where *m* is an integer. To conveniently process the experimental data, we use Equation (22) to calculate the phase variation difference between the two ESOPs.

**Figure 8:**
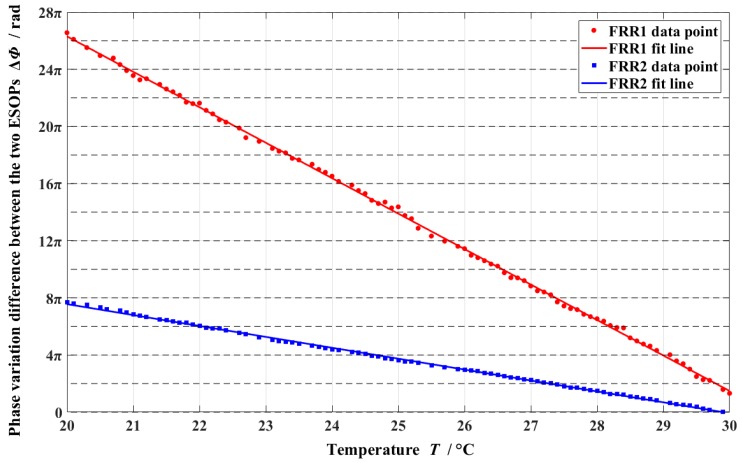
Experimental results of the phase variation differences of FRR1 and FRR2 in the range of 20 °C to 30 °C (ESOP: eigenstate of polarization and FRR: fiber ring resonator).

Figure 8 depicts the polarization crosstalk of the two FRRs at 20 °C–30 °C. After linear fitting, the relation between the phase variation differences of the two FRRs and temperature can be obtained as follows:

(23) $$\Delta \Phi_{1}=-7.798\cdot \left(T-30.598\right)+2\pi m_{1},$$

(24) $$\Delta \Phi_{2}=-2.401\cdot \left(T-29.895\right)+2\pi m_{2},$$

where *m*_1_ and *m*_2_ are integers. From Equations (23) and (24), the polarization crosstalk periods of the two FRRs are

(25) $$\frac{2\pi dT}{d\Delta \Phi_{1}}=-0.8058\;{}^{\circ}C,$$

(26) $$\frac{2\pi dT}{d\Delta \Phi_{2}}=-2.6171\;{}^{\circ}C.$$

Thus, one cycle of crosstalk is observed in FRR1 per temperature change of approximately 0.8058 °C, and one cycle of crosstalk is observed in FRR2 per temperature change of approximately 2.6171 °C. The temperature stability of FRR2 is 3.2478 times that of FRR1, which indicates that the use of hybrid PM-PCF FRR can effectively suppress the polarization crosstalk.

Substituting the aforementioned experimental results of Equations (25) and (26) into Equations (19) and (20), respectively, we can obtain

(27) $$\frac{d\Delta n_{\mathrm{eff}}}{dT}=-4.8091\cdot 10^{-7}/{}^{\circ}C,$$

(28) $$\frac{d\Delta {n^{\prime }}_{\mathrm{eff}}}{dT}=-0.3712\cdot 10^{-7}/{}^{\circ}C.$$

Therefore, the experimental results depict that the temperature stability of a PM-PCF is 12.955 times that of a Panda-PMF. Table 3 presents the theoretical simulation and experimental results of the birefringent temperature coefficients of the two optical fibers in 20 °C–30 °C.

## 5. Conclusions

We theoretically analyzed and experimentally verified the polarization phase shift errors of a Panda-PMF and a PM-PCF, as caused by the thermally induced birefringence changes. Using the finite element numerical calculation method, it was proved that the temperature stability of a PM-PCF was 11.268 times that of a Panda-PMF. In the experiment, it was observed that the temperature stability of FRR2 (composed of a PM-PCF and a Panda-PMF with a length ratio of 3:1) as compared with that of FRR1 (composed of a Panda-PMF) was increased to 3.2478 times. According to the experimental results, the temperature stability of a PM-PCF was 12.955 times that of a Panda-PMF. The results exhibit that a PM-PCF can be used to optimize an FRR and to effectively suppress polarization fluctuation. With the improvement of fiber fusion technology, the application of PM-PCF in RFOG has great potential, which can greatly improve the environmental adaptability of the RFOG.

## Figures and Tables

**Table 1:** Geometrical parameters of the optical fibers. SAPs = stress applying parts.

Panda-PMF	PM-PCF
Core diameter = 6 μm SAPs diameter = 36 μm Distance between SAPs and core = 28 μm	Small hole diameter = 4 μm Large hole diameter = 7.2 μm Minimum spacing of small hole Λ = 6.7 μm Deviation of small hole Δ = 0.57 Number of layers of small hole = 7
Acrylate diameter = 250 μm Cladding diameter = 125 μm	

**Table 2:** Material Parameters of the Optical Fibers.

Material	Acrylate	SiO_2_	SiO_2_ + GeO_2_	SiO_2_ + B_2_O_3_
Thermal expansion coefficient *α* (10^−6^/K)	80	0.55	0.62	2.42
Poisson’s ratio *ν*	0.38	0.17	0.17	0.20
Young’s modulus *E* (GPa)	0.6	72.4	72.4	49.2

**Table 3:** Birefringent temperature coefficient of fibers in the range of 20 °C–30 °C.

Thermal Coefficient	Theoretical Simulation Results	Experimental Results
dΔ*n*_eff_/d*T*	−4.62 × 10^−7^/°C	−4.8091 × 10^−7^/°C
dΔ*n*’_eff_/d*T*	−0.41 × 10^−7^/°C	−0.3712 × 10^−7^/°C
(dΔ*n*_eff_/d*T*)/(dΔ*n*’_eff_/d*T*)	11.268	12.955
